# Retrospective analysis on the safety of 5,759 times of bedside hyperthermic intra-peritoneal or intra-pleural chemotherapy (HIPEC)

**DOI:** 10.18632/oncotarget.7622

**Published:** 2016-02-23

**Authors:** Lili Liu, Ning Zhang, Jie Min, Haichuan Su, Hongmei Wang, Dongxu Chen, Li Sun, Hongwei Zhang, Wei Li, Helong Zhang

**Affiliations:** ^1^ Department of Oncology, Tangdu Hospital, The Fourth Military Medical University, Xi'an, China; ^2^ Department of Gastroenterological Surgery, Xijing Hospital, The Fourth Military Medical University, Xi'an, China

**Keywords:** bedside HIPEC, safety, intra-pleural, intra-peritoneal

## Abstract

The current study was designed to analyze safety of the bedside hyperthermic intra-pleural or intra-peritoneal chemotherapy (HIPEC) from September 2007 to July 2015. Total of 5,759 times of bedside HIPEC in 985 cases of malignant pleural or peritoneal carcinomatosis were analyzed. Of them, 1,510 times was given to 315 cases of malignant pleural effusion, while 4,249 times was performed in 402 patients with malignant ascites and 268 patients without ascites (total 670 patients for peritoneal carcinomatosis). In average, patients with pleural effusion was given 5 times bedside HIPEC and stayed in the hospital for 6.7 days; while patients with peritoneal carcinomatosis was given 6 times of HIPEC and stayed in the hospital for 6.5 days. Overall HIPEC-associated mortality was zero. Overall HIPEC-associated incidence of side effect in the intra-pleural HIPEC was 2.0%. Specifically, 0.6% was pneumothorax, 0.3% was cytotoxic agent-induced pleural inflammation, 0.5% was pain at puncture location, and 0.3% was failure of HIPEC procedure. Overall HIPEC-associated incidence of side effect in the intra-peritoneal HIPEC was 2.4%, i.e., failure of HIPEC procedure in 1.3%, pain at puncture location was 0.5%, cytotoxic agent-induced peritoneal inflammation was 0.1%, intestinal obstruction was 0.1% and intestinal perforation was 0.07%. These findings indicated that bedside HIPEC applied in the current study is safe to be performed by a Physician or Oncologist under local anesthesia at a patient's bedside. The procedure is easy to perform and well-tolerated by the patients with late stage cancer or post-surgery recurrent cancer.

## INTRODUCTION

Since the first report of hyperthermic intra-peritoneal chemotherapy (HIPEC) by Spratt in 1980 [1], HIPEC has been gradually recognized as an effective adjuvant therapy to peritoneal carcinomatosis (PM). Nevertheless, HIPEC has been predominantly used as intra-surgery procedure in an operating room either under open abdomen or closed abdomen right after surgical resection of abdominal tumors. The strategy of complete cytoreductive surgery (CCRS) plus HIPEC was mainly developed and standardized by PH Sugarbaker [2] and this combination has achieved significant improvement in patient's quality of life and prolongation of survival rate in many patients or even cure in certain diseases such as peritoneal pseudomyxoma [3]. However, this type of intra-surgery HIPEC has the following limitations. First, it has to be performed in an operation room. Thus, it is invasive and expensive. Second, it is limited to do only once for a patient who is suitable to receive surgical resection of a tumor, but not for those patients who are in late stage or with post-surgery recurrent cancer. Third, cytotoxic effect of the chemotherapeutic drugs may affect tissue repair and wound healing if it is perfused into the peritoneal cavity right after surgery, and thus, may result in high prevalence of side effects such as infection, peritonitis, or even pancreatitis.

Therefore, we have been using a novel bedside HIPEC device since 2007. This new HIPEC procedure has the following advantages: 1). It can be used in a patient's bedside or a treatment room. 2). It can be accomplished under local anesthesia. 3). It is easy to operate and can be performed by a Physician or an Oncologist without help from a Surgeon or Anesthesiologist. 4). It can be used for patients in late stage or with recurrent cancer after surgical resection of solid tumors. 5). Multiple times of HIPEC can be applied for the same patient.

The aim of this study was to retrospectively analyze safety of 5,759 times of bedside HIPEC performed in the Department of Oncology, Tangdu Hospital, affiliated to The Fourth Military Medical University, for 985 patients with malignant pleural effusion or peritoneal carcinomatosis with or without ascites.

## RESULTS

### Demographic information of the patients with malignant pleural effusion

Total 315 patients with malignant pleural effusion were given 1,510 times bedside intra-pleural HIPEC therapy. Of them, 230 were male (73.0 %) and 85 were female (27.0%); aged from 23- 90 years old (median age: 63); and 192 (61.0%) were late stage primary cancer and 123 (39.0%) were post-surgery recurrent patients. By the cell type of the primary cancer, 160 cases (50.8 %) were lung cancer; 50 cases (15.9 %) were breast or gynecological cancer; 25 cases (7.9 %) were carcinoma of gastroenterological system; 20 cases (6.3 %) were liver cancer; 15 cases (4.8 %) were malignant thymoma; and 45 cases (14.3 %) were miscellaneous types of cancer (Table 1).

**Table 1:** Clinical characteristics of the patients

	Intra-pleural HIPEC	Intra-peritoneal HIPEC
Total # of cases	315	670
Primary	192 (61.0%)	408 (60.9%)
Recurrent	123 (39.0%)	262 (39.1%)
Sex		
Male	230 (73.0%)	305 (45.5%)
Female	85(27.0%)	365 (54.5%
Total HIPEC times	1510	4249
Age (year)		
Youngest	23	27
Oldest	90	82
Average	63	59
Times of HIPEC		
1-3	80 (25.4%)	140 (20.9%)
4-6	185 (58.7%)	350 (52.2%)
≥7	50 (15.9%)	180 (26.9%)
Average	5	6
Hospitalization (day)		
≤4	10 (3.2%)	45 (6.7%)
4-7	235 (74.6%)	460 (68.7%)
>7	70 (22.2%)	165 (24.6%)
Average	6.7	6.5

### Demographic information of the patients received intra-peritoneal HIPEC

Total 670 patients were given 4,249 times of intra-peritoneal HIPEC therapy. Of them, 305 (45.5 %) were male and 365 (54.5 %) were female patients; aged from 27-82 years old (median age: 59); 405 (60.4 %) cases were with malignant ascites and 265 (39.6 %) were without ascites; 408 (60.9%) were primary cancer and 262 (39.1%) were post-surgery recurrent patients. By the cell type of the primary cancer, 200 cases (29.9 %) were stomach cancer; 190 cases (28.4 %) were ovarian cancer; 105 cases (15.7 %) were liver cancer; 30 (4.5 %) were pancreatic cancer; 30 (4.5 %) were colon cancer; 15 cases (2.2 %) were peritoneal pseudomyxoma; and 100 cases (14.9 %) were miscellaneous types of cancer (Table 1).

### Analysis on safety of the intra-pleural HIPEC

**Figure 1:**
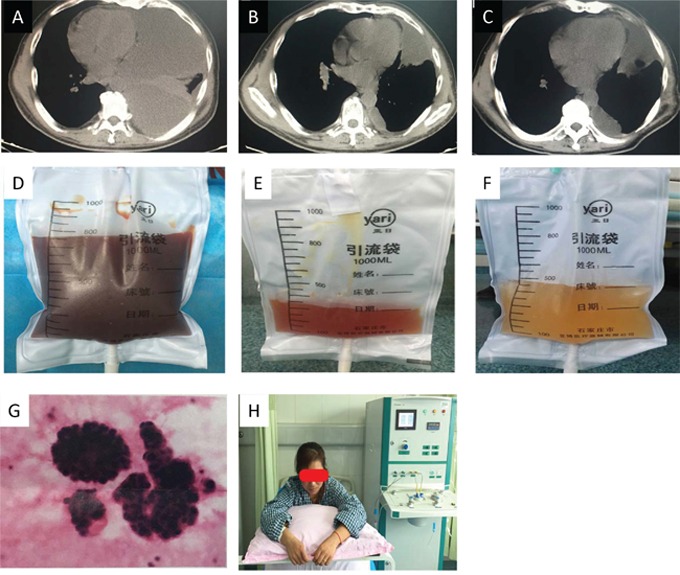
Recurrent pleural effusion after surgical resection of lung adenocarcinoma **Panels A, B, C**: CT images before HIPEC (A), after one therapeutic course (B), and two therapeutic courses (C), respectively. **Panels D, E, F**: Color change of the pleural effusion lavage before HIPEC (D), after one therapeutic course (E), and two therapeutic courses (F), respectively. **Panel G:** Cytologic examination of the lavage fluid before HIPEC demonstrated adenocarcinoma. **Panel H**: Patient sat up and received HIPEC therapy for one hour.

A representative case of hyperthermic intra-pleural chemotherapy was presented in Figure 1. As shown in Figure 1A, CT image presented large amount of pleural effusion on the left, which was bloody effusion (Figure 1D) and contained adenocarcinoma cells (Figure 1G). After 1-2 course of HIPEC therapy, amount and color of bloody effusion was significantly reduced in most of cases as evidenced by chest CT examination (Figure 1B and 1C) and collection of the lavage fluid (Figure 1E and 1F).

As shown in Table 2, of the 325 patients who received intra-pleural HIPEC treatment, 80 patients (25.4 %) received 1-3 times of bedside HIPEC, 185 patients (58.7 %) received 4-6 times, and 50 patients (15.9 %) received at least 7 times HIPEC treatment. In average, patients with malignant pleural effusion received 5 times HIPEC therapy and stayed in the hospital for 6.7 days. Of them, 10 patients (3.2 %) stayed in the hospital less than 4 days; 235 patients (74.6 %) stayed for 4-7 days; and 70 patients (22.2 %) stayed longer than 7 days.

**Table 2:** Morbidity of intra-pleural HIPEC-associated side effect

Side effect	N	%
Pneumothorax	10	0.6%
Pleural inflammation	5	0.3%
Pulmonary embolism	0	0%
Bleeding	0	0%
Infection of puncture point	3	0.2%
Pain at puncture point	7	0.5%
HIPEC procedure failure	5	0.3%
Death	0	0%
Total	30	2.0%

Of the 1,510 times HIPEC treatment, overall prevalence of side effect was only 2.0%. Of that, 10 times (0.6 %) was pneumothorax; 5 times (0.3 %) cytotoxic agent-induced pleural inflammation; 3 times (0.2%) infection of puncture points; 7 times (0.5 %) pain at the puncture points; and 5 times (0.3 %) failed to perform HIPEC procedure. There was no pulmonary embolism, heavy bleeding or death in any case.

### Analysis on safety of the intra-peritoneal HIPEC

**Figure 2:**
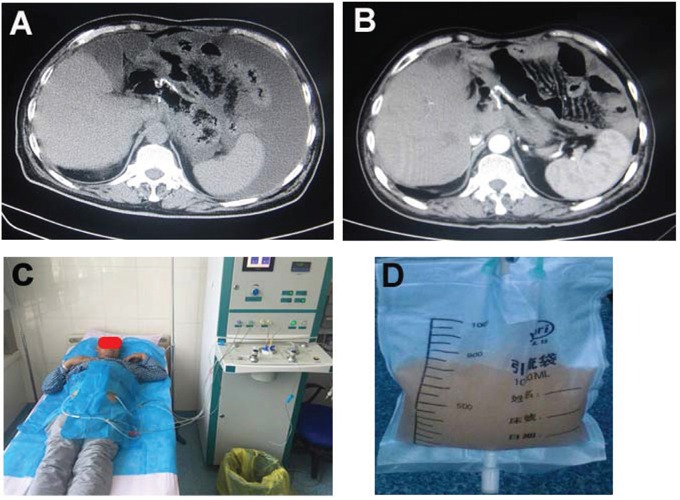
Gastric carcinomatosis and ascites **Panel A**: Before HIPEC therapy. **Panel B**: One week after 2 times HIPEC (cisplatin: 37.5mg/m^2^ each time on day 1 and 3). **Panel C**: Patients with HIPEC therapy at supine position. **Panel D**: Chylous ascites which was washed out from the peritoneal cavity.

**Figure 3:**
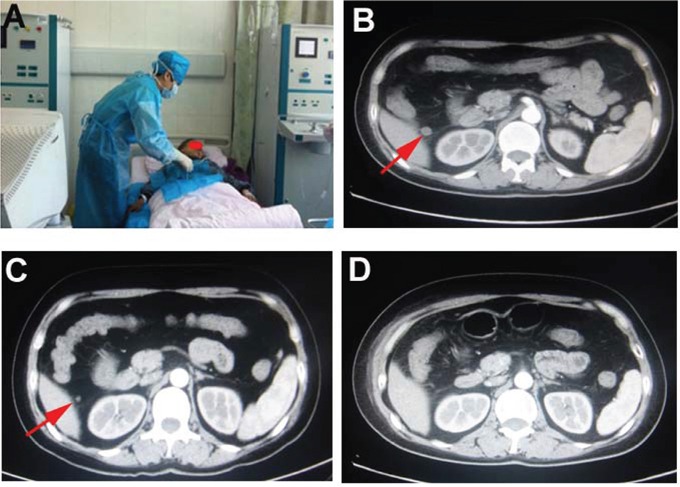
Lymph node metastasis of ovarian cancer **Panel A**: Patient with HIPEC therapy at supine position. **Panel B**: Enlarged lymph node (arrow) before HIPEC therapy. **Panel C**: One week after HIPEC therapy, the lymph node was significantly reduced in size (arrow). **Panel D**: Two weeks after HIPEC therapy, the lymph node was further reduced in size.

Two representative cases of hyperthermic intra-peritoneal chemotherapy were presented as Figure 2 and Figure 3. Figure 2 was a case of malignant ascites after surgical resection of gastric cancer. Figure 2A and B indicated significant reduction of the malignant ascites after 2 times of HIPEC therapy. The patient was at supine position (Figure 2C) and chylous fluid was washed out from the abdominal cavity before HIPEC therapy (Figure 2D). Figure 3 was a case of lymph node metastasis after surgical resection of ovarian cancer and received 2 therapeutic courses of HIPEC (Figure 3A). Figure 3B, 3C and 3D indicated an enlarged lymph node before HIPEC therapy (Figure 3B), and a significant reduction of the lymph node after 1 and 2 therapeutic courses (Figure 3C and 3D, respectively).

Of the 670 patients who received intra-peritoneal bedside HIPEC, 140 patients (20.9 %) received 1-3 times, 350 patients (52.2 %) received 4-6 times, and 180 patients (26.9 %) received at least 7 times of HIPEC treatment. In average, 6 times of HIPEC was received in the patients with peritoneal carcinomatosis, and patients stayed in the hospital for an average of 6.5 days (Table 3). Of them, 45 patients (6.7 %) stayed in the hospital less than 4 days; 460 patients (68.7 %) stayed for 4-7 days; and 165 patients (24.6 %) stayed longer than 7 days.

**Table 3:** Morbidity of intra-peritoneal HIPEC-associated side effect

Side effect	N	%
Bleeding	0	0%
Infection of puncture point	5	0.2%
Pain at puncture point	35	0.8%
Peritoneal inflammation	5	0.1%
Intestinal obstruction	5	0.1%
Intestinal perforation	3	0.07%
HIPEC procedure failure	55	1.3%
Death	0	0%
Total	103	2.4%

Of the 4,249 times of intra-peritoneal bedside HIPEC therapy, overall prevalence of side effect was 2.4 %. Of that, 55 times (1.3 %) failed to perform HIPEC procedure, 35 times (0.5 %) was pain at the puncture location, 5 times (0.1 %) cytotoxic agent-induced peritoneal inflammation, 5 times (0.2%) infection at the puncture points, 5 times (0.1 %) intestinal obstruction, and 3 times (0.07 %) intestinal perforation. However, there was no severe bleeding or death in any case (Table 3).

## DISCUSSION

While surgical resection is the first choice of therapy for a visible tumor, many malignant tumors are unsuitable for surgery due to either they are in late stage when they are diagnosed or they are recurrent tumors following initial surgical resection, and most of cases, if not all, they are invisible and diffused in the pleural or peritoneal cavities. Pleural or peritoneal carcinomatosis or metastasis after surgical removal of a primary tumor is one of the major factors contributing to high mortality rate in patients with variety types of carcinomas including lung cancer, gastrointestinal cancer or ovarian cancer. For such patients, especially patients with malignant pleural or peritoneal effusion, hyperthermic intra-pleural or intra-peritoneal chemotherapy (HIPEC) appears to be an efficient adjuvant therapy in addition to systemic chemotherapy or immunotherapy.

Since September 2007, a novel procedure of bedside HIPEC has been conducted in our facility. In the current study, we retrospectively analyzed safety of this novel bedside HIPEC treatment. Of the HIPECs conducted in malignant pleural effusion, the HIPEC-associated mortality rate was zero, and overall incidence of side effects associated with HIPEC procedure, including failure of HIPEC procedure, pneumothorax, cytotoxic agent-associated pleural inflammation, and pain at the puncture location, was very low. Moreover, there was no severe side effect such as pulmonary embolism or severe bleeding. Similarly, of the HIPECs conducted in peritoneal carcinomatosis with or without malignant ascites, HIPEC-associated mortality was zero and overall incidence of HIPEC-associated side effects was such as HIPEC procedure failure, pain at the puncture locations, cytotoxic agent-induced peritoneal inflammation, intestinal obstruction, and intestinal perforation was very low. These findings suggested bedside HIPEC applied in the current study is safe.

In 1980, Spratt was the first to describe a clinical delivery system for intra-peritoneal hyperthermic chemotherapy to treat a recurring peritoneal pseudomyxoma [1], followed by Paul H. Sugarbaker from USA [2, 4] and Fujimoto from Japan [5] who reported that combined application of cytoreductive surgery plus intra-peritoneal hyperthermic perfusion during or right after the surgery could effectively treat patients with peritoneal carcinomatosis. The main principles of this strategy are: treating the visible tumor of peritoneal seeding with complete cytoreductive surgery (CCRS), and immediately after the surgical resection, treating the remaining invisible malignant seeding cells in the peritoneal cavity with HIPEC. Since then, the strategy of CCRS + HIPEC has been used as classical HIPEC therapy for intra-peritoneal carcinomas in the last 27 years by surgeons from worldwide including USA, Europe, Australia and China [3, 4, 6–8]. CCRS plus intra-operational HIPEC significantly improved cancer progress free survival and overall survival rate in patients with peritoneal carcinomatosis such as peritoneal pseudomyxoma and mesotheliomas [9–11]. Nowadays, strategy of CCRS plus intra-surgical HIPEC has been considered as standards for the following peritoneal diseases [3, 12, 13]. 1). Recognized as the gold standard for the treatment of peritoneal pseudomyxomas and peritoneal mesotheliomas; 2). Considered as a standard of care for the treatment of colorectal peritoneal carcinometastsis; 3). HIPEC is in the evaluation phase for gastric and ovarian peritoneal carcinomatosis.

However, there are several limitations in the intra-operational HIPEC. *First*, it is invasive and expensive. *Second*, it has to be done during or right after the surgical resection and patient has to be under systemic anesthesia, which may increase mortality or morbidity of complication. In this regard, in the French Registry containing 1,290 patients treated in 25 centers, the postoperative mortality rate was 4% and grade 3 or 4 complications (according to the National Cancer Institute Common Toxicity Criteria) occurred in 34 % of the patients [14]. Similarly, in an Australian study, the postoperative mortality rate was 3% and severe morbidity rate was 43% [15]. *Third*, most of recurrent peritoneal carcinomatoses are not suitable for surgical resection, but the patient may seek a palliative therapy. For these patients, intra-operational HIPEC is not practical. In contrast, the novel HIPEC applied in the current study can be used in a patient's bedside and for palliative or adjuvant purpose. Furthermore, the patient was in consciousness during the whole HIPEC procedure in that only topical anesthesia was used to establish the sealed hyperthermic circulation. More importantly, the mortality that was directly derived from the bedside HIPEC procedure *per se* was zero in both hyperthermic intra-pleural and intra-peritoneal chemotherapy, and overall prevalence of side effects was only 2.0% for the intra-pleural HIPEC, or 2.4% overall prevalence of side effects for the intra-peritoneal HIPEC procedure, suggesting the bedside HIPEC applied in the current study is safe and easy to be tolerated by patients.

HIPEC techniques are heterogeneous in terms of selecting chemotherapeutic drugs and their concentration, composition and volume of the perfusion, circulating duration, and temperature etc. Different combination of these parameters may result in different consequence of the HIPEC therapy. Currently, there are two trends worldwide for HIPEC: one uses mitomycin C over 60-90 min at 41°C with closed-abdomen technique, and the other uses oxliplatin (± irinotecan) over 30-40 min at 43°C with an open-abdomen technique [3]. In the current study, however, we chose to use bedside HIPEC using a device that has the following two functions: “One-way Washing” function and “Hyperthermic Circulation” function. The “One-way Washing” was performed prior to the circulation of hyperthermic saline containing chemotherapeutic agent. This washing procedure benefits the patient and dramatically improves therapeutic effect by the following mechanisms. 1). Wash out bloody solution containing cancer cells and tissue; 2). Flowing water may have adhesiolysis effect on fibrin or sanctuary of cancer seeding cells; 3). Better distribution of the chemotherapeutic agent inside the body cavity by reducing protein concentration in the circulating solution.

In the current study, chemotherapeutic agent for HIPEC was determined based on the cell type of the primary cancer and its sensitivity to the drug. Intra-body cavity temperature was projected to be 41 ± 1°C. Volume of the hyperthermic circulation was approximately 1500 mL for intra-pleural HIPEC and 3000-5000ml for intra-peritoneal HIPEC, and duration of HIPEC was 60 min. Typically, one therapeutic course was consisted of 3 times HIPEC, which was performed every other day or every 3 days due to the thermo-tolerance of human tissues [16].

In the current study, hyperthermic intra-peritoneal chemotherapy was conducted for the patients not only with ascites, but also without ascites. The latter patients were those who had surgical resection, but post-surgical pathology indicated that they were very likely or definitely had lymph node metastasis. Preventive HIPEC was initialized in these patients within 3-4 weeks after surgery in order to reduce possibility of peritoneal carcinomatosis and ascites. Our experience demonstrated it was plausible and safe to perform HIPEC in the patients without ascites.

The HIPEC device used in the current study was manufactured by Xi'an Good Doctor Medical Science and Technology, Xi'an, China and there are following advantages compared to the HIPEC devices used for conventional CCRS + HIPEC in the surgery. 1). It is minimally invasive and patient stays consciousness for the whole period of HIPEC procedure in that topical anesthesia is used to establish the HIPEC circulation. 2). It can be used in a patient's bedside or a non-surgical treatment room, and thus, it is cost effective. 3). Multiple times of HIPEC treatment can be performed for the same patient. 4). It can be used for not only intra-peritoneal HIPEC, but also intra-pleural HIPEC. 5). Most importantly, HIPEC-derived mortality rate is zero and much less incidence of side effect. In this regard, the morbidity of overall side effect in the current study was 2.0% in the 1,510 times of intra-pleural HIPEC and 2.4% in the 4,249 times of intra-peritoneal HIPEC. In contrast, the open-surgery HIPEC-associated overall morbidity of side effect was as high as 14-56% [12, 13].

Limitation of the HIPEC used in the current study, however, was the occurrence of HIPEC procedure failure due to unable to establish a circulation (most cases are in the patients without ascites) or blockade of circulation. The major causes of the failure were either tissue adhesion following surgery, or accidental intestinal perforation in the patients without ascites, or insufficient lavage of the malignant effusion. In addition, tissues such as omentum and mesentery may also cause blockade of the hyperthermic circulation during the procedure of the intra-peritoneal HIPEC. However, with improvement of puncturing skill under the guidance of type B ultrasound and using larger gauge needles, aforementioned problems will be significantly reduced.

Taken together, bedside HIPEC can be used as an adjuvant or palliative therapy for malignant pleural or peritoneal carcinomatosis. While most of HIPEC have been conducted during or right after the surgery in the operation room, a bedside HIPEC can be applied under topical anesthesia by a Physician or Oncologist in a patient's room or a non-surgical treatment room. By retrospectively analyzing over 5000 times of bedside HIPEC treatment, the study demonstrated that bedside HIPEC-derived mortality was zero and overall HIPEC-associated morbidity of side effect was very low in both intra-pleural HIPEC and intra-peritoneal HIPEC. Findings of the current study suggest that this novel HIPEC procedure is safe to be used for multiple times in a patient and effective as a palliative therapy for late stage or post-surgery recurrent cancer patients.

## MATERIALS AND METHODS

### Patients

Patients with malignant pleural effusion and peritoneal carcinomatosis (PM) with or without ascites, who were hospitalized in The Department of Oncology, Tangdu Hospital, Affiliated to the Fourth Military Medical University, Xi'an, China, from September 2007 through July 2015, were enrolled into the current study. A written Consent Form was obtained from each patient. Study Protocol and Design were approved by the Ethics Committee of the Tangdu Hospital, The Fourth Military Medical University, and all procedures were performed in accordance with the 1964 Helsinki Declaration and its later Amendments.

Diagnosis of malignant pleural effusion or PM was confirmed by histology or cytology. Patients with pleural effusion were late stage lung cancer or metastatic cancers of breast, liver or stomach cancer. Patients for PM were either with ascites or without ascites, and miscellaneous cell types of intra-peritoneal carcinoma. None of the patients had cardiovascular disease, brain metastasis, active intra-pleural or intra-peritoneal bleeding, or other contraindication for hyperthermic chemotherapy.

### Procedure of hyperthermic intra-pleural chemotherapy

Patients were in sitting position with arms and head on a supporting table in front of the patients (Figure 1H). Location and amount of pleural effusion was examined, and two puncture points of In-flow and Out-flow for sealed hyperthermic circulation were then determined with the ultrasound. After routine skin disinfection and local anesthesia, routine puncture was performed with the needle and catheters in a disposable package provided by the manufacture of GD-HIPEC device (Xi'an Good Doctor Medical Science and Technology Ltd. Inc., Xi'an, China). The In-flow port was then connected to the tube of the HIPEC machine followed by thorough lavage of the malignant effusion with pre-warmed saline by choosing “One-way washing” mode on the HIPEC machine. Approximately 2000 mL of pre-warmed saline was used for each case to lavage the bloody effusion in the pleural cavity. When the red color of the bloody effusion turned into clear saline, the Out-flow port was connected to the HIPEC machine and a sealed circulation of the hyperthermic (41 ± 1°C) saline containing chemotherapeutic agent was then initiated by choosing “Circulation” mode on the machine. After 60 min of sealed circulation of HIPEC, 600 mL of the solution was drained out from the pleural cavity and the rest of the heated saline with chemotherapeutic drug was left inside the pleural cavity.

### Procedure of hyperthermic intra-peritoneal chemotherapy

Different procedures were applied to patients with or without ascites, respectively. As shown in Figure 2 and 3, patients were given intra-peritoneal HIPEC at supine position. For the patients with ascites, similar procedure as the chest HIPEC was applied. Briefly, two puncture points were determined by ultrasound followed by “One-way” lavage of the bloody and thick intra-peritoneal effusion. Afterwards, sealed hyperthermic (41 ± 1°C) circulation was performed with 1500 mL heated saline containing chemotherapeutic drug. For the patients without ascites, the first puncture point for In-flow port was determined with ultrasound by selecting a point where abdominal organs were avoided. A puncture was performed under local anesthesia as described above. Total 500 mL pre-warmed saline was then slowly injected in order to confirm the needle was at intra-peritoneal cavity without injuring any abdominal organs. Once it was confirmed that the needle was in the peritoneal cavity, additional 2500 - 4500 mL pre-warmed saline was then perfused into the abdominal cavity by the GD-HIPEC machine (Xi'an Good Doctor Medical Science and Technology Ltd. Inc., Xi'an, China). The second puncture point for Out-flow port was then determined by ultrasound on the opposite site of the abdomen. After connecting the Out-flow port to the HIPEC machine, sealed circulation with hyperthermic (41 ± 1°C) saline containing chemotherapy drug was then established as described above. Intra-peritoneal HIPEC was also performed for 60 min followed by leaving approximately 1500 mL solution in the abdomen cavity.

### Chemotherapeutic drugs used for HIPEC

Chemotherapeutic drug for HIPEC was selected in accordance with sensitivity of the primary carcinoma to the drugs. Following drugs were used: cisplatin, 5-fluorouracil, mitomycin C, Adriamycin, and cyclophosphamide. Normally, one kind of aforementioned chemotherapeutic drug was used for a patient, which was injected into the sealed HIPEC circulation system. Total dose was determined by body weight, divided by 3 and given at 2-3 separate HIPEC procedures (1/2-1/3 total dose was given at each time HIPEC), which was performed every other day or every 3 days for one complete therapeutic course.

### Data collection and analysis

In addition to basic demographic information, the following data or information was collected or recorded. Times of receiving HIPEC therapy; type of chemotherapeutic drug used; side effects associated with HIPEC *per se*, and days of hospitalization.
